# Demands and resources of a long-standing bring-your-dog-to-work program: a constant comparative analysis

**DOI:** 10.3389/fpubh.2025.1576360

**Published:** 2025-06-10

**Authors:** Braxton Schieler, Niwako Ogata, Leanne O. Nieforth

**Affiliations:** ^1^Human-Animal Partnerships and Interactions Lab, Department of Comparative Pathobiology, Center for the Human-Animal Bond, College of Veterinary Medicine, John Martinson Honors College, Purdue University, West Lafayette, IN, United States; ^2^Department of Veterinary Clinical Sciences, Center for the Human-Animal Bond, College of Veterinary Medicine, Purdue University, West Lafayette, IN, United States; ^3^Human-Animal Partnerships and Interactions Lab, Center for the Human-Animal Bond, Comparative Pathobiology, College of Veterinary Medicine, Purdue University, West Lafayette, IN, United States

**Keywords:** job demands-resources model, workplace wellness, mental health, bring-your-dog-to-work, work-life balance, organizational belonging

## Abstract

**Introduction:**

Given the evidence that companion animals may provide social and emotional support to their human counterparts, some companies have begun offering bring-your-dog-to-work programs in an effort to reduce employee strain and improve workplace wellness outcomes. The purpose of this qualitative study was to investigate how a long-standing bring-your-dog-to-work program at a large midwestern university veterinary college, the Dogs at Work Program, impacted the workplace well-being of program participants.

**Methods:**

A total of *n* = 11 staff and faculty members who participated in the program completed semi-structured interviews about their experiences. Interviews were analyzed using the constant comparative technique.

**Results:**

Constant comparative analysis revealed four themes situated within a job demands-resources theoretical framework: (1) Dogs providing emotional support as a resource, (2) Dogs providing social support as a resource, (3) Increased responsibility as a demand, and (4) Lack of adherence to program rules as a demand. Results indicated that bringing pet dogs into the workplace was viewed positively because the dogs provided an impetus for pleasant breaks from stressful work, improved work-life balance, and helped to develop and enhance social relationships. On the other hand, participants also mentioned that dogs could be a distraction from workplace productivity, especially if they were poorly behaved.

**Discussion:**

Well-enforced policies for dog activity and behavior are critical to ensure that dogs at work do not diminish productivity or upset some employees. Nevertheless, bring-your-dog-to-work-programs appear to show promise in terms of reducing strain and may be linked to improved mental health outcomes.

## 1 Introduction

Approximately 77% of working adults in the United States have experienced stress at work in the past month (1). Workplace stress may have multiple negative impacts on employee mental health including emotional exhaustion, reduced motivation, reduced productivity, and an increased intention to quit. This trend should alarm employers, as employees are likely to respond to “persistent, inescapable stressors” by giving up [(2), p. 132]. This phenomenon, known as quiet quitting, occurs when an employee reduces effort and ceases “to be fully committed to [their] job and [does] just enough to meet the requirements of [their] job description” [(3), p. 9]. Moreover, recent research indicates that workplace stressors such as high workload and issues with coworkers have significant positive correlations with burnout, depression, and suicidal ideation (4, 5). Thus, workplace stressors appear to impact the totality of an individuals’ well-being, rather than just their satisfaction with and well-being at work.

The specific stressors experienced at work vary across industries and individuals; however, two common stressors include challenges related to workload and work-life balance as well as lack of organizational belonging. With respect to workload, professionals from numerous disciplines cite long hours and high responsibilities as significant stressors (6–8). However, while the sheer volume of work may cause stress, the issue of workload appears more complex. Specifically, stress associated with workload may be impacted by poor separation between work and life, especially as more individuals are working from their homes. Whereas a physical office building serves as both a physical and mental boundary between one’s place of work and home, this natural delineator does not exist when working from home, making it easier for employees to transition between work and leisure activities and increasing expectations for employees to always be ready to complete a new task (9). Addressing challenges caused by poor work-life balance is of critical importance; those with poor work-life balance are substantially more likely to experience psychological distress (10).

In addition to challenges related to workload and work-life balance, employees may feel stressed in workplaces where they feel that they do not belong—both in terms of being supported by and socially identifying with their organizations (11, 12). When individuals feel their “work organization values their contributions and cares about their well-being,” they are more likely to be engaged in and satisfied with their jobs [(13), p. 101, (14)]. Employees are more likely to view their jobs positively and less likely to experience workplace stress when their employers take steps to understand and meet their employees’ needs (2, 12, 15). Yet critically, over a quarter of workers surveyed in the American Psychological Association’s 2023 Work in America Survey reported feeling loneliness or isolation at work, indicating that employees may lack a sense of belonging and being supported at work (1). When employees feel unsupported by their organizations, they are more likely to perceive stressful situations at work as threatening because they lack social resources in their colleagues and superiors to help them navigate these situations (2, 16).

One way to understand how workplace challenges affect employees is through the job demands-resources model (JD-R) (17). In this theoretical framework, demands are “those physical, social, or organizational aspects of the job that require sustained physical and mental effort and are therefore associated with certain physiological and psychological costs” [(17), p. 501]. Practically, job demands are the stressors of work that increase emotional exhaustion, such as stressful workloads and poor work-life balance (18). Resources, on the other hand, are “those physical, psychological, social, or organizational aspects of the job that may do any of the following: be functional in achieving work goals, reduce job demands at the associated physiological and psychological costs; stimulate personal growth and development” [(17), p. 501]. When employees feel that they lack organizational support and a sense of belonging, they are expressing the lack of a key occupational resource that may contribute to reduced motivation and heightened cynicism (18). In some cases, this imbalance of demands and resources can result in burnout—“a prolonged response to chronic emotional and interpersonal stressors on the job” [(19), p. 397]. Managing burnout is critical as numerous studies have found links between burnout and concerns with mental health (e.g., suicidal ideation), employees’ intent to leave the organization and overarching workplace culture (4, 20–23).

Accordingly, many employers are looking to add to the resources available to help their workers’ manage workplace demands including offering employee resource groups to foster tight-knit community and providing employee assistance programs to help employees process and manage various work or life stressors (24–28). One such resource is bring-your-pet-to-work programs. Interacting with a companion animal may have stress-reducing effects (29–32). Given these stress-reducing effects, recent research has examined the potential impact pets in the workplace might have on reducing workplace stress and burnout (33–37). One study found that employees who brought their dogs to work were more dedicated to their work and had lower turnover intention, higher home-work interface, and stronger feelings of control at work compared to those who did not bring their dogs (38). Additionally, pets may encourage employees to take short breaks wherein employees can recover from stressful encounters before continuing to work (33, 39). Pets may also help to make the workplace more pleasant and less stressful by facilitating social interactions between coworkers that may otherwise not have happened (40, 41). Taken together, these results show strong potential for pets in the workplace to act as a critical resource to offset stressful demands of practice. However, to date, most studies have focused on the presence of pets in work-from-home settings or have compared the perspectives of employees’ working at companies that do and do not offer pet-friendly policies. To the authors’ knowledge, one study to date has evaluated the impacts of a long-standing dogs-in-the-workplace program, but no study has done so within the context of a veterinary college (42). The purpose of this study was to explore if and how a long-standing bring-your-dog-to-work program impacts the workplace well-being of program participants.

## 2 Materials and methods

This manuscript reports qualitative data gathered from semi-structured interviews from a larger, mixed-methods study. Semi-structured interviews allow participants flexibility to emphasize aspects of their experiences that are most salient to them (43). The University Institutional Review Board approved this study as exempt (IRB #2024-196).

### 2.1 The dogs at work program

Founded in 1999, the Dogs at Work (DAW) Program is an initiative available to faculty and staff (e.g., veterinarians, researchers, veterinary nurses, hospital staff and postdoctoral fellows) of the veterinary college of a large midwestern university. Dogs must be evaluated by a veterinary behaviorist for good behavior according to a modified version of the American Kennel Club’s Canine Good Citizen Test prior to being allowed in their owners’ offices. The availability of the program is limited to specific roles and office locations within the college where dogs are least likely to cause challenges in the ordinary functioning of the college. Given the long-standing nature of the program, it is an ideal context to study the impacts of the presence of pets in the workplace; the owners’ perceptions of the program and the associated outcomes are based on extensive experience rather than an immediate reaction to novel conditions.

### 2.2 Participants

To recruit participants, an email was sent to faculty and staff within the university’s veterinary college inviting them to complete an online survey via Qualtrics experience management software. Reminder emails were sent once a week for 4 weeks. The survey was open for 1 month. The survey contained a consent form followed by a series of validated measures related to mental health, social health, and workplace well-being, as well as a question asking if participants would be interested in being contacted for a follow-up interview. Survey results are reported elsewhere (44). Participants with office-certified dogs who expressed interest in being interviewed were sent an additional email inviting them to participate in a semi-structured interview.

At the time of this study, there were 40 office-certified dogs in the college. A total of 11 individuals who completed the initial survey, had at least one office-certified dog and indicated interest in participating in a follow-up interview were recruited to be interviewed. Current literature supports utilizing a similar sample size for qualitative interview studies as this tends to provide enough data to obtain theoretical saturation and a “deep understanding of a phenomenon” without becoming too dense for meaningful analysis to be practical (45–48). Many different roles within the veterinary field were represented among the interview participants including veterinarians, veterinary technicians, administrative staff, and researchers. Given the small population for the current study, additional demographics on participants and their dogs were not collected to protect participant confidentiality (49).

### 2.3 Semi-structured interview procedure

Semi-structured interviews were conducted via Zoom for approximately 30 min by author LN. All interviews were recorded and later transcribed. Semi-structured interview questions included: (1) “What does a day with your dog in the office look like?” (2) “Why do you take your dog to work with you?” (3) “What are the benefits of having your dog with you at work?” (4) “What are the challenges of having your dog with you at work?” (5) “Is there anything else you would like to share about your experiences?” Consistent with best practices for semi-structured interviews, follow-up questions for clarification were asked when needed for clarity or when the researcher wanted to learn more about a participants’ perspective (43).

## 3 Analysis

Results were analyzed using a grounded theory approach (47). More specifically, a constant comparative analysis was conducted (50). Constant comparative analysis uses an iterative process of comparing themes between individual interviews (i.e., emic) and comparing those themes to established theoretical frameworks (i.e., etic) (51). Authors BS and LN initially read the transcribed individual interviews and created codes for each topic and theme discussed (52). Next, the authors condensed the total number of codes by rereading the interviews and looking for overlap between themes. Finally, to maximize agreement on the codes and themes developed, the authors compared the coded interview themes to established theoretical frameworks with the goal of selecting the best theoretical framework to situate and guide the themes identified within the semi-structured interviews. Authors BS and LN met regularly to refine and align themes and codes and to discuss relevant theories found in literature. This iterative process continued until no new themes emerged—attaining theoretical saturation—and the themes were well-situated within a single theoretical framework, the JD-R model (17, 47).

## 4 Results

Constant comparative analysis of participant interviews revealed four themes that were situated within the JD-R model: Theme 1. Emotional support as a resource, Theme 2. Social support as a resource, Theme 3. Additional responsibilities as a demand, and Theme 4. Lack of awareness as a demand.

### 4.1 Theme 1: emotional support as a resource

Participants identified five ways that bringing their dogs to work provided emotional support: the provision of empathy and understanding, lack of judgment, a source of humor, physical contact, and the opportunity for breaks during the day.

#### 4.1.1 Emotional understanding as an emotional support resource

Several participants reflected that their dogs had a unique ability to “just know” human emotions, often providing care and support when those emotions were negative. One individual commented that “if somebody is crying, he [the dog] is like Velcro to them… he [the dog] has that intuition.” Another added: “[My dog knows] who to go to when they are upset… if I’m upset, she’ll go to me. But if there’s… staff members upset she’ll go to them. She’ll know which… [people] are having a bad day.”

#### 4.1.2 Lack of judgment as an emotional support resource

Beyond perceiving the emotions of their owners and their owner’s colleagues, dogs were valuable emotional support resources because they provided care without judgment. One owner was grateful that “[dogs] give you that unconditional love.” Another participant elaborated: “They’re always happy seeing you; they do not care how moody you are. For them, it’s like the most important thing is you be there, right? The rest they do not care.”

In addition to being grateful for the judgment-free emotional support their dogs provided them, participants were appreciative that their dogs—in contrast to students or colleagues—were present without making demands:

For me it was probably more like… walking into my office and there was something there to greet you that wasn’t a student asking you for something… someone’s excited to see you and they’re not gonna, like, ask for anything.

#### 4.1.3 Breaks as emotional support resources

One of the most significant benefits of the DAW program for participants was the opportunity to take purposeful breaks: “It was nice to… come into my office after maybe a difficult client or I usually do my [heavy-hearted work such as] euthanasia, at the end of the day… [so] it was nice to be able to like have them on the way home.” Whether because of client interactions, euthanasias, or other aspects of veterinary work, numerous interviewees reflected that having their dog was helpful for decompressing after stressful encounters:

The benefits to me [are] … being able to like take a break from some of the things that we do. Because… this job can be very stressful—just stepping outside, like cooling off, taking a second to… be out in the yard … you know, like, we deal with euthanasia and sad cases all the time. So just like taking a second to… be with your own dog after that I think is super important.

More specifically, breaks could be helpful because they promoted exercise and broke up the monotony of long workdays:

The days I have to spend in my office… looking at medical records all day long… it’s not very fun. It’s nice to have a little distraction … and something that gets me up out of my chair to go outside and take a little walk … have lunch outside or just sit for a little bit.

While breaks occasionally afforded opportunities for staff members to take an extended rest from work by taking their dog outside and getting exercise, at times they were also simple pauses where the dog’s presence could be a comfort and a decompressing agent after a stressful encounter:

He’s a good stress reliever … after like a phone call where it didn’t go well or there was some type of situation that was like not really needed. You just kind of look at him and he’s just like right there saying ‘OK, lady’ … it’s always nice just to see him kind of be there just as an extra support.

While these comments reaffirm the manifold stressors veterinary staff experience, they demonstrate that the ability to “get away for 5 minutes” is helpful for reducing tensions compared to when the dog is not in the office and “it takes… longer to decompress.”

#### 4.1.4 Physical contact as an emotional support resource

The ability to touch, hold, or pet their dog was of value to many participants: “A dog that lets you pet it… you feel those endorphins release, and you are like ‘yes.’” It may be that in some cases, the benefits to staff of taking breaks with their pets were caused by the physical contact shared during those breaks: “honestly, sometimes there are tougher days… something does not go as you plan and you know, just petting the dog as you write something… it just makes it so much better… having the 5 minute puppy cuddle in the office.”

#### 4.1.5 Humor as an emotional support resource

Dogs may engage in “cute” or funny behaviors which can be a source of levity and humor during an otherwise stressful day:

[My dog] knows when it’s the end of the day. It’s really funny kind of watching him… when he knows it’s time to go home… he’s by the door just waiting. Which is great. And then as soon as I open up the door, he wants to bound out.

Like physical contact, this humorous aspect of dogs in the workplace may be another mechanism through which taking breaks from work is an effective stress reliever:

[When you take] the mental break from work and then you can go back to work and you’re like ‘oh, I feel so happy—I just watched my dogs have so much fun…’ you just feel happier and more energized.

### 4.2 Theme 2: social support as a resource

DAW program participants identified three ways in which their dogs acted as a social support resource: an enhancer of work-life balance a tool for building community, and as evidence of support from administration.

#### 4.2.1 Improved home-work interface as a social support resource

One of the most stressful aspects of veterinary work is the long hours and lack of work-life balance (4, 10, 53–55). The ability to bring one’s dog to work helped address these challenges and in some cases even made dog ownership possible: “I would not have a dog with my schedule if I had to leave it home all day.” Many participants felt that being allowed to bring their dog to work helped them enjoy their pet without feeling guilty about leaving it at home during their long hours: “It’s a vet school, right? You can have a dog, and you can still… have a working life… without feeling bad getting a puppy and the puppy sits at home alone.” Similarly, one participant expressed that the program freed her from the guilt of enjoying a social life after working hours:

I had dogs when I was in vet school, and I always felt guilt… because you left them… you felt like you couldn’t go do anything afterwards because you just have to go [home] to your pets. Now the pets are with me at work, so I don’t feel bad for… going to my hobbies in the evenings.

There was also a practical advantage to being able to bring dogs to work, especially during the puppy phase:

It’s convenient to puppy train [and] potty train a dog when you are still working, and [it] doesn’t interfere when you’re working at all. And then you can actually stay longer at work because you don’t feel like you need to rush back home.

The ability to bring one’s dog into the office appears to reduce work-related stress via enhanced work-life balance and to improve productivity because of this reduced tension.

#### 4.2.2 Community building as a social support resource

One of the primary benefits of the DAW program was the sense of community in the office that the participants felt the program provided. Dogs in the office functioned as a social bridge that facilitated the development of relationships as one participant describes with their new students:

These were… first year students and so they didn’t really know us that well. It was first semester and we as instructors… we didn’t really have an established relationship, and I think that [having my dog] kind of helped put us on the same level… the students didn’t feel as intimidated.

In some cases, as with students, dogs helped “break the ice” in developing important relationships. In other cases, dogs helped to foster a sense of community by initiating social interactions that may otherwise not have happened. One participant described how their dogs helped to develop a relationship with a member of the janitorial staff:

The other thing that [my dogs] love is their cleaning people at night… [my dog] would hear the cleaning person come and he would literally leave the office [to meet him] … then [the cleaning person] would just… pet them on the floor and … sit there and then we would talk about his pet… it’s such a good way of interacting with people… oftentimes people neglect like the cleaning people… but I would literally chat with him every day… but I would never chat with him if I didn’t have the dogs.

For some participants, the ability to bring their dog to work provided a sense of meaning and purpose because it offered an opportunity to show care for colleagues and students: “sometimes like I know [the students are] having a bad week, or I know they have a stressful exam, so sometimes I’ll bring him… the day before or the day after.” In a similar vein, one dog owner said, “we go in the back just to… make everyone’s day a little bit brighter.” A few individuals discussed how their dog helped serve as an emotional buffer when the negative emotions of others grew intense or difficult: “It was really nice to have something to buffer [emotions] because I’m not an emotional person… when people cry in my office… I’m like ‘good job… here’s a tissue.’ And so [my dog is] like the emotional one.” One participant described an example of this with a struggling student:

There was a student who was having a really bad day. [She] had two exams, felt like she failed them. I found her crying in the hallway. And she basically was just like ‘Is [Dog Name] in your office’ and I was like ‘yeah [Dog Name] is in my office… [Dog Name] was all over her and just loved on her for an hour. And then she said that she felt so much better afterward. And I was like, “yeah, see, that’s why she’s here.”

Between colleagues, the presence of dogs in the office helped to create a sense of community where colleagues could depend on each other in stressful situations and rely on their colleagues’ dogs as a resource: “anytime… my coworkers are like having a bad day… they will like get him out.” As one example, a participant shared:

If [one of my coworkers] gets done with like a [difficult phone call] they’ll come over. ‘[Name], I have to tell you all about this.’ And of course [my dog] will go right up to them and just kind of let them play with them, pet him or what not… I can tell as they’re telling the story, they’re not as stressed as they were [when] they came in.

#### 4.2.3 Support from administration as a social support resource

Numerous participants expressed gratitude for the opportunity to bring their dog to the office: “I just want to say that I really appreciate being able to bring an animal and I do not know if I would have stayed here if that wasn’t an option.” Another echoed this sentiment, “I see it… as a privilege… being able to bring my dog.” Participants appreciated that dogs were screened for safety before being allowed into the office: “I really appreciate this opportunity… that I can bring dogs in and they are getting screened for safety and everything… in my previous institution, they did not let us bring [a] dog and that was hard.” These comments demonstrate that the DAW program leaves employees feeling grateful and supported.

### 4.3 Theme 3: increased responsibility as a demand

Interview analysis revealed two ways in which DAW program acted as a demand by increasing responsibilities: concern for colleagues, and productivity and privacy challenges.

#### 4.3.1 Concern for colleagues as an increased responsibility demand

Nearly all participants indicated that bringing their dog to work resulted in a heightened awareness of the perceptions of their dogs among colleagues which could at times be stressful. In many cases, this involved practical concerns, like considering where to take one’s dog to use the restroom: “I always… take them elsewhere to potty before I take them to a courtyard… if you take your dog to a courtyard, which is like people eating lunch… that may not be nice.” In other cases, dog owners needed to accommodate to each other when office spaces were shared:

I think that would cause a little bit of anxiousness if, you know, I had an office mate that… maybe… my dog and their dog didn’t get along and so we’d have to coordinate when they came in, or maybe they weren’t like happy with having a dog in the office.

More succinctly, another dog owner said, “not everybody wants to have a dog around all the time.” To this end, DAW program participants recognized that they needed to be respectful and careful around their colleagues who may not share their affinity for dogs:

I know some people are afraid of dogs, or just don’t like them… I’m trying to be respectful… because at the end of the day there are more people without dogs than people with dogs… so if you don’t respect certain boundaries, it’s going to be, in the long run, bad for people.

Colleagues’ needs and desires are categorized as a demand because of the extra responsibility for DAW program participants to concern themselves with, which they would not have had without the program.

#### 4.3.2 Productivity and privacy as increased responsibility demands

Dogs in the workplace appear to afford their owners more breaks. However, while these breaks can function as resources that reduce strain, they can also be seen as distractions that limit productivity. One dog owner, for example, discussed the difficulty of training a dog while on the job: “When he was a puppy, I needed to train him… to stay quiet in my office. He was not easy, especially with a dog with anxiety and… he would cry.” Finding an appropriate place to take the dog during the day was also a struggle: “Sometimes it was… finding a place to take her… we did not have a good dog park kind of area for our personal pets.” In other cases, dogs in the office served as an unspoken invitation for eager colleagues to say hello which was sometimes seen as an impediment to productivity:

It can be a challenge if… you have multiple people kind of coming into your space [to see your dog] and you know you’re trying to get some work done and it’s, you know, sometimes those days just seem to be… ‘Oh my gosh, I can never get a minute to do something.’

### 4.4 Theme 4: lack of adherence to program rules as a demand

Many participants indicated that the structure provided by the clear behavior guidelines and regulations of the DAW Program were a major strength of the program. In some cases, dog behavior remained a concern. One participant said that “there are some dogs that have [barked at my dog] through the gate.” Another added, “We’ve had situations… just walking down the hallway and the dog has busted through their gate.” In general, dog owners echoed the sentiment that a one-time-certification was likely insufficient for differentiating a well-behaved dog from a poorly behaved dog: “[Your dog becomes office-certified] and then… you are good for life… I’m not sure that should be the case.” Another participant added, “I think we need a better way of differentiating who [has a well-behaved dog] and who does not have one… some people say that they have [well-behaved dogs] and they are really not [well-behaved].”

## 5 Discussion

The purpose of this qualitative study was to understand whether and how the DAW Program influences the workplace well-being of program participants. Constant comparative analysis of 11 semi-structured interviews with faculty and staff who participated in the program identified four themes situated within a JD-R framework.

### 5.1 Theme 1: emotional support as a resource

There are several unique emotional demands of working in a veterinary college. For example, euthanasia-related responsibilities are significantly associated with secondary trauma and compassion fatigue (56). Furthermore, in the context of a veterinary college, many staff members work with students, which carries an additional potential for compassion fatigue as employees share the professional and academic burdens of their students in addition to their own professional demands (57). Even without considering these more emotionally demanding aspects of working in a veterinary college, veterinary staff still face stringent demands including long working hours, poor work-life balance, and unreasonable client expectations (8, 53–55, 58–60). These strains are not only stressful, they are also significantly associated with burnout (4).

Participants in the current study referred to each of these demands in their interviews, giving support to the notion that working in a veterinary college is stressful. Encouragingly, however, participants’ comments seem to indicate that their pets’ presence at work provided the emotional support needed to cope with these demands. This finding is in line with research that found that companion animals were a significant source of social support and stronger attachment to one’s companion animal predicted stronger feelings of emotional support (61). DAW program participants may have experienced enhanced emotional support when their dogs were with them at work because of the high attachment they had to their pets. An interesting question for further exploration is whether working in close proximity to one’s personal pet *increases* attachment and thus provides increasingly stronger emotional support over time as the strength of the human-animal bond increases.

Many participants reflected that their dogs seemed to intuitively understand their emotions. Indeed, domestic dogs have “tremendously complex abilities to perceive… emotional expressions” and to functionally respond to this information [(62), p. 1]. More specifically, the advantage of a dog in providing emotional support appears to be that the dog can interpret their owner’s emotions without casting judgment or creating expectations and demands. One study found that dog owners experienced lower cardiovascular reactivity when performing a stressful arithmetic task in the presence of their dog compared to the presence of their spouse (29). In this case, the dog appears to provide comfort via proximity without making evaluative judgments as a human supporter might. In the workplace context, dogs may be able to provide more support than colleagues because of this lack of judgment. Another study found that veterinary nurses reported higher levels of burnout in response to patient suffering and death when social support *increased* (63). The authors speculated that this phenomenon occurred because the well-intentioned support of colleagues merely served to underscore problems without offering solutions. Pets in the workplace may be able to provide more effective social support than coworkers because they do not offer judgment and do not reemphasize the individual’s sad or stressful experiences.

Results indicated that participants took more short, restorative breaks from work when their dogs were with them. According to conservation of resources theory, individuals have limited energy and “cognitive attention” to handle workplace stressors (64). When employees take short ‘microbreaks’ to socialize or relax, they recharge their cognitive resources and reset after experiencing stress at work (64). One researcher found that even looking at or petting one’s dog can act as a “pleasant micro-break capable of restoring the regulatory resources necessary for effective performance” [(33), p. 9]. Participants indicated that having their dog with them at work provided the motivation for taking microbreaks such as walking or petting their dog(s) after a stressful conversation with a client. In this sense, dogs may motivate or even force employees to take salutary breaks to recover their resources and decompress in ways that—due to high workplace demands—they may feel unable to do on their own (54). In another vein, there may be specific ways that pets provide emotional support—for example, via physical contact or humorous interactions—which make them an especially beneficial way to take a break from work (32). Further research on the specific mechanisms through which interactions with pets reduce stress and provide emotional support in the workplace is necessary.

### 5.2 Theme 2: social support as a resource

Although psychological distress among veterinarians is higher than in the general US population, “not enough can be said about the importance of work-life balance” which is “the number one predictor of high levels of well-being, low burnout, and good mental health” among veterinarians [(10), p. 956]. It is noteworthy, then, that nearly all participants in the current study highlighted improved home-work interface as one of the most significant benefits of the DAW program. Several individuals mentioned that they would not be able to manage the responsibilities of dog ownership without the program. Further, a few participants mentioned that the program enhanced their life outside of work by providing them time for hobbies or social activities that they could not have participated in if they had needed to spend their precious few hours at home attending to their dogs. Given the adverse mental health effects of the long hours that some veterinary professionals work and the demands they experience, programs like the DAW program that enhance life outside of work are of critical importance (10, 55, 65).

Beyond providing social support by enhancing work-life balance, dogs in the workplace appeared to promote improved relationships between colleagues. Participants in the current study indicated that their dogs both facilitated conversations with people they might otherwise not have interacted with (e.g., janitorial staff), and helped to begin more long-lasting friendships between students and faculty and between colleagues. This finding is in line with previous research suggesting that dogs can act as a “social catalyst”; strangers and acquaintances are significantly more likely to begin an interaction with a dog present than without, and these interactions have the potential to evolve into close friendships (66, 67). Since team-focused workplace cultures with high degrees of trust and collaboration are associated with higher organizational commitment and less stress for employees, dogs in the workplace may prove to be valuable assets in improving workplace wellbeing (66, 68).

Finally, several participants indicated their gratitude for the program. Employees are more likely to feel supported by their organizations when initiatives to support them are developed voluntarily; for example, increasing wages by choice rather than because of an increase in the minimum wage (13). Since the DAW program is voluntarily maintained by administration with the intent of supporting employees, it likely increases employees’ perceived organizational support (POS). Higher levels of POS are associated with numerous positive outcomes including increased job satisfaction, higher organizational commitment and reduced stress (13, 34, 36). While the current study only investigated dog owners participating in the DAW program, one study found that POS improved in pet-friendly workplaces even among non-dog-owners (42). While the present study only interviewed dog-owning program-participants, it may be that the mere opportunity to bring a dog to work may generate a feeling of being supported which can reduce stress regardless of whether employees take advantage of the program or not. Furthermore, gratitude may elicit numerous personal benefits including reduced stress, depression, and feelings of loneliness (69, 70).

### 5.3 Theme 3: additional responsibility as a demand

Some participants mentioned that dogs in the workplace could create distractions and diminish productivity. This finding is also seen in other studies about pets in the workplace (37, 40, 71). One study found that two competing processes often occur during workplace distractions (72). On the one hand, distractions deplete limited self-regulatory resources needed for productivity as employees must turn their attention to the distraction, attend to it as necessary, and return to the previous task. This negative process will indeed challenge productivity. Conversely, distractions can help to fulfill a need for belongingness; small conversations and interactions with coworkers—and perhaps with their dogs—can help an employee feel valued as a member of the whole. These competing processes may explain why comments about the DAW program were largely positive and emphasized the social support that dogs in the workplace helped to provide. Interacting with a colleagues’ dog was more likely to enhance feelings of belonging than to be viewed as a negative distraction. On the other hand, mere “nuisance issues such as barking” that did not contribute to belongingness were viewed as disturbances that limited productivity [(37), pp. 73, 85]. In and of itself, taking breaks to interact with a dog may be a restorative benefit for employees. However, without firmly established guidelines for dog behavior, occasional breaks may become frequent disturbances that hinder productivity for employees who enjoy dogs, and may also impede on the privacy of colleagues who may not like dogs.

### 5.4 Theme 4: lack of adherence to program rules as a demand

Several studies about pets in the workplace reinforce the importance of clear rules and guidelines for dog behavior (40, 42). The DAW program addresses these concerns by requiring that dogs be evaluated for good behavior before coming to the office. Participant comments indicated that the program guidelines had a positive impact on dog owners’ conscientiousness of their dogs’ behavior—they commented that the program was a privilege and referenced the need for dogs to be well-behaved. However, participants still mentioned instances of aggressive or distracting dog behavior. In these instances, the demand is not a lack of guidelines, but a lack of enforcement mechanisms for the guidelines that exist. Given that dog behavior was a concern in veterinary contexts where many employees are comfortable around dogs, it is especially critical that clear enforcement mechanisms are present if bring-your-dog-to-work programs are to be implemented in more general workplaces where employees may not share this relative ease.

### 5.5 Limitations and future directions

When interpreting the results of the current study, several limitations should be considered. The study only interviewed individuals who participated in the program and regularly brought their dogs to work. Dog-owning individuals appeared to appreciate the program largely because of their attachment to their dogs and the social and emotional support they provided. Future studies should investigate the perceptions and experiences of employees who do not have a dog or who do not regularly bring their dog to work. More specifically, research should investigate whether and how non-dog owners benefit from pet-friendly workplaces and the extent to which they view dogs at work to be a hindrance to productivity. Furthermore, this study was conducted within the veterinary college of a large midwestern university. While this context was useful given the long-established nature of the DAW program and important given the unique demands encountered by veterinary professionals, future studies should consider additional workplace contexts. It is worth investigating whether the same benefits are experienced in a context where more employees may not be used to dogs, may be afraid of dogs, and where dog owners may be less conscientious of the need to manage their dogs’ behavior. Finally, future bring-your-dog-to-work programs should explore enforcement strategies for program guidelines. Where workplace productivity is concerned, it is not sufficient to state that dogs should be well-behaved; strategies must be in place to handle cases where they are not.

## 6 Conclusion

This constant comparative analysis investigated the perceived influence of a longstanding bring-your-dog-to-work program on workplace well-being of program participants. Using a job demands-resources framework, participants generally tended to view access to their dog at work as a resource more so than as a demand. Participants felt that their dogs provided emotional support to cope with stressors in the workplace by offering non-judgmental support and encouraging their owners to take more breaks from work. Further, dogs in the workplace provided social support by promoting work-life balance and helping to develop a sense of community in the workplace. While these social advantages are primarily for dog owners who participate in the program, we hypothesize that they may also extend to others in the workplace, who may benefit by participating in conversations facilitated by dogs and who may have increased perceived organizational support simply by working in a pet-friendly office. Future research is warranted to explore how bring-your-pet-to-work programs impact the well-being of employees who do not participate in the program. Demands mentioned by participants centered on dogs’ behavior being distracting or inappropriate for the workplace. These concerns underscore not only the need for clear guidelines, but also for enforcement mechanisms of said guidelines. In conclusion, findings suggest that a bring-your-dog-to-work program may improve employees’ perceived well-being, especially in professions with intense demands and high propensity for burnout.

## Data Availability

The datasets presented in this article are not readily available because the data may be identifying to the participants. Requests to access the datasets should be directed to Leanne Nieforth, lniefort@purdue.edu.
